# CSDE1 Intracellular Distribution as a Biomarker of Melanoma Prognosis

**DOI:** 10.3390/ijms25042319

**Published:** 2024-02-15

**Authors:** Alberto Indacochea, Tanit Guitart, Aram Boada, Vicente Peg, Ariadna Quer, Hafid Laayouni, Laura Condal, Pablo Espinosa, Jose Luis Manzano, Fátima Gebauer

**Affiliations:** 1 Centre for Genomic Regulation (CRG), The Barcelona Institute of Science and Technology, Dr Aiguader 88, 08003 Barcelona, Spain; indacochea@recerca.clinic.cat (A.I.); tanit.guitart@crg.eu (T.G.); pablo.espinosa@crg.eu (P.E.); 2 Dermatology Department, Hospital Universitari Germans Trias i Pujol, Institut d’investigació Germans Trias I Pujol, Universitat Autònoma de Barcelona, 08916 Badalona, Spain; aboada.germanstrias@gencat.cat (A.B.); lcondalrovira@gmail.com (L.C.); 3 Pathology Department, Vall d’Hebron University Hospital, Passeig Vall d’Hebron 119-129, 08035 Barcelona, Spain; vicente.peg@vallhebron.cat; 4 Pathology Department, Hospital Universitari Germans Trias I Pujol, Institut d’Investigació Germans Trias I Pujol, Universitat Autònoma de Barcelona, 08916 Badalona, Spain; 5 Institut de Biologia Evolutiva (UPF-CSIC), Department of Medicine and Life Sciences, Universitat Pompeu Fabra, Dr Aiguader 88, 08003 Barcelona, Spain; hafid.laayouni@upf.edu; 6 Barcelona Beta Brain Research Center, Pasqual Maragall Foundation, C/Wellington 30, 08006 Barcelona, Spain; 7 Medical Oncology Department, Catalonian Institute of Oncology, (ICO), Hospital Germans Trias I Pujol, 08916 Badalona, Spain; jmanzano@iconcologia.net; 8 Department of Medicine and Life Sciences, Universitat Pompeu Fabra, Dr Aiguader 88, 08003 Barcelona, Spain

**Keywords:** CSDE1, melanoma, biomarker, RNA-binding protein, cytoplasmic–nuclear ratio, prognosis

## Abstract

RNA-binding proteins are emerging as critical modulators of oncogenic cell transformation, malignancy and therapy resistance. We have previously found that the RNA-binding protein Cold Shock Domain containing protein E1 (CSDE1) promotes invasion and metastasis of melanoma, the deadliest form of skin cancer and also a highly heterogeneous disease in need of predictive biomarkers and druggable targets. Here, we design a monoclonal antibody useful for IHC in the clinical setting and use it to evaluate the prognosis potential of CSDE1 in an exploratory cohort of 149 whole tissue sections including benign nevi and primary tumors and metastasis from melanoma patients. Contrary to expectations for an oncoprotein, we observed a global decrease in CSDE1 levels with increasing malignancy. However, the CSDE1 cytoplasmic/nuclear ratio exhibited a positive correlation with adverse clinical features of primary tumors and emerged as a robust indicator of progression free survival in cutaneous melanoma, highlighting the potential of CSDE1 as a biomarker of prognosis. Our findings provide a novel feature for prognosis assessment and highlight the intricacies of RNA-binding protein dynamics in cancer progression.

## 1. Introduction

Melanoma is one of the most aggressive forms of skin cancer, accounting for 80% of skin cancer deaths. In the last 15 years, inhibitors against frequent mutations in the BRAF–MAPK pathway and antibodies against immune checkpoint blockers have dramatically increased patient survival [1,2]. However, about 50% of patients do not respond or become refractory to treatment [3]. In addition, melanoma is a highly heterogeneous disease, complicating clinical diagnosis [4]. The high diversity of melanoma lesions, and the fact that tumors of barely a few millimeters in depth have the capacity to metastasize, has stirred considerable efforts to identify reliable biomarkers for diagnosis, treatment choice and prognosis. These include not only pathological features, but gene or protein expression signatures, miRNAs, genomic mutations and epigenomic modifications [5,6,7,8]. Curiously, RNA-binding proteins (RBPs) have not generally been considered in the melanoma biomarker field. RBPs are one of the largest protein families in the cell, with more than 1500 members [9]. RBPs control all steps in the life of mRNA and can modulate virtually all cancer hallmarks through shaping the cancer cell proteome and regulating the activity of important cellular RNP machineries such as the spliceosome or the ribosome [10,11]. RBPs are frequently mutated in human genetic disease, although their frequency of somatic mutations in cancer is reduced compared to other protein families such as transcription factors [12,13,14]. They are emerging as important modulators of cell plasticity, therapy resistance and immunomodulation, fueling an increased interest in RBPs as druggable targets for cancer prevention and treatment [15,16,17,18]. 

We have previously found that the RBP CSDE1 (also called UNR) behaves as an oncoprotein in melanoma [19]. CSDE1 promotes invasion and metastasis by downregulating the steady-state levels of mRNAs encoding tumor suppressors (e.g., PTEN) and promoting the synthesis of the EMT factors Vimentin and RAC1 [19]. In addition, CSDE1 mediates immune evasion of melanoma cells by modulating the levels of the phosphatase TCPTP and thereby decreasing STAT1 phosphorylation [20]. CSDE1 has been involved in other tumor types, often displaying context-specific functions [21]. Similar to melanoma, CSDE1 maintains the invasive phenotype of colorectal, pancreatic and lung cancer cells [22,23,24,25], while in squamous cell carcinoma, paraganglioma and pheochromocytoma, CSDE1 behaves as a tumor suppressor [26,27]. Given the extensive involvement of CSDE1 in cancer and its important roles in melanoma progression, we aimed to test its value as a biomarker of prognosis. Here, we first generate a monoclonal antibody optimal for immunohistochemistry and benchmark the antibody against the Human Protein Atlas (HPA) dataset by assessing CSDE1 expression in 14 healthy human tissues. We then evaluate CSDE1 expression in 149 whole tissue sections including benign nevi, and melanoma primary tumors and metastasis. We find that CSDE1 expression is heterogeneous in healthy tissues and, contrary to expectations from an oncoprotein, its levels decrease with melanoma malignancy. Interestingly, the intracellular distribution of CSDE1 changes with tumor stage. Indeed, we find that CSDE1 cytoplasmic/nuclear ratio is an indicator of progression free survival. These results highlight the potential of CSDE1 as a predictive biomarker of prognosis in melanoma.

## 2. Results

### 2.1. Design of the Study

The study consisted of an exploratory analysis of 149 non-matched specimens, including 50 nevi, 50 primary melanoma tumors and 49 metastatic melanoma samples. All nevi were intradermic, 68% of which were from females and 68% from subjects younger than 58 years old. Primary and metastatic samples were more diverse. Primary tumors were gender balanced (50%), most of which (78%) belonged to individuals older than 58 years and were taken at various body locations. Histopathological classification and other clinical features (Breslow, ulceration and mitotic index) are indicated in Table 1.

Regarding metastatic samples, 65% belonged to males, 78% were from patients older than 58 years and the vast majority (82%) derived from primary cutaneous melanoma, although for 12% the primary was unknown. A total of 41% were found to be mutated for BRAF and 14% for NRAS, and nearly 50% were classified as Stage III at diagnose. Importantly, 78% of samples were naive for treatment at the time of collection. Properties of metastatic samples are summarized in Table 2. The levels and intracellular localization of CSDE1 were measured in these samples to evaluate its value as a marker of prognosis.

### 2.2. Generation of a Suitable anti-CSDE1 Monoclonal Antibody

**Figure 1:**
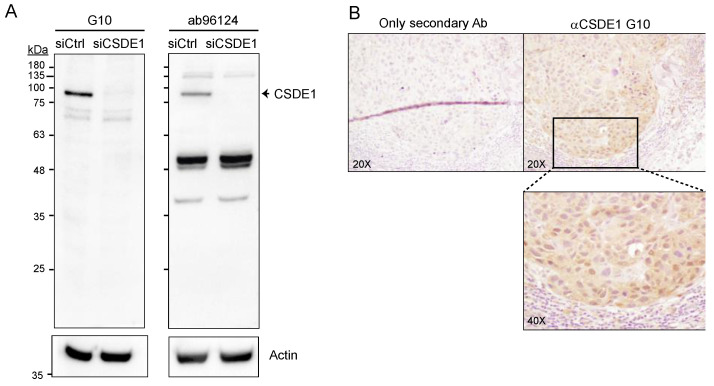
Characterization of the G10 monoclonal antibody. (**A**) Comparison of the G10 (left panel) and ab96124 (right panel) reactivities in melanoma SK-Mel-147 cells depleted (siCSDE1) or not (siCtrl) of CSDE1. Actin is shown as loading control. Full blots of actin are shown in Figure S1A. (**B**) Immunohistochemistry of a nodular melanoma. Staining with secondary antibody alone is shown as control. Magnification is indicated on the lower left corner.

There are currently several rabbit polyclonal antibodies that have been used in immunohistochemistry (IHC) after affinity purification against CSDE1 (see https://www.proteinatlas.org, accessed on 20 November 2023; and refs [19,22,23]). The polyclonal nature of the antibodies, and the fact that they need to be affinity purified, imposes limitations for routine clinical usability. In order to generate a source of ready-to-use antibody useful for the community without limitations of quantity or purity, we obtained a mouse monoclonal antibody in collaboration with Eurogentec. This antibody, which we term G10, specifically detects human CSDE1 via Western blot, as shown by a prominent signal in melanoma cells that disappears after depletion of CSDE1 (Figure 1A, left panel). The antibody is more specific than the Abcam ab96124 previously used to assess CSDE1 levels in patient samples in several reports including ours [19,22,23], highlighting limitations of those works. This is evidenced by (i) the absence of the strong contaminating bands that are detected with the ab96124 antibody (Figure 1A, compare left and right panels), and (ii) a clear and stronger reduction in signal upon CSDE1 depletion observed with immunocytochemistry (Figure S1B). Importantly, the antibody efficiently detects CSDE1 in IHC (Figure 1B).

**Figure 2:**
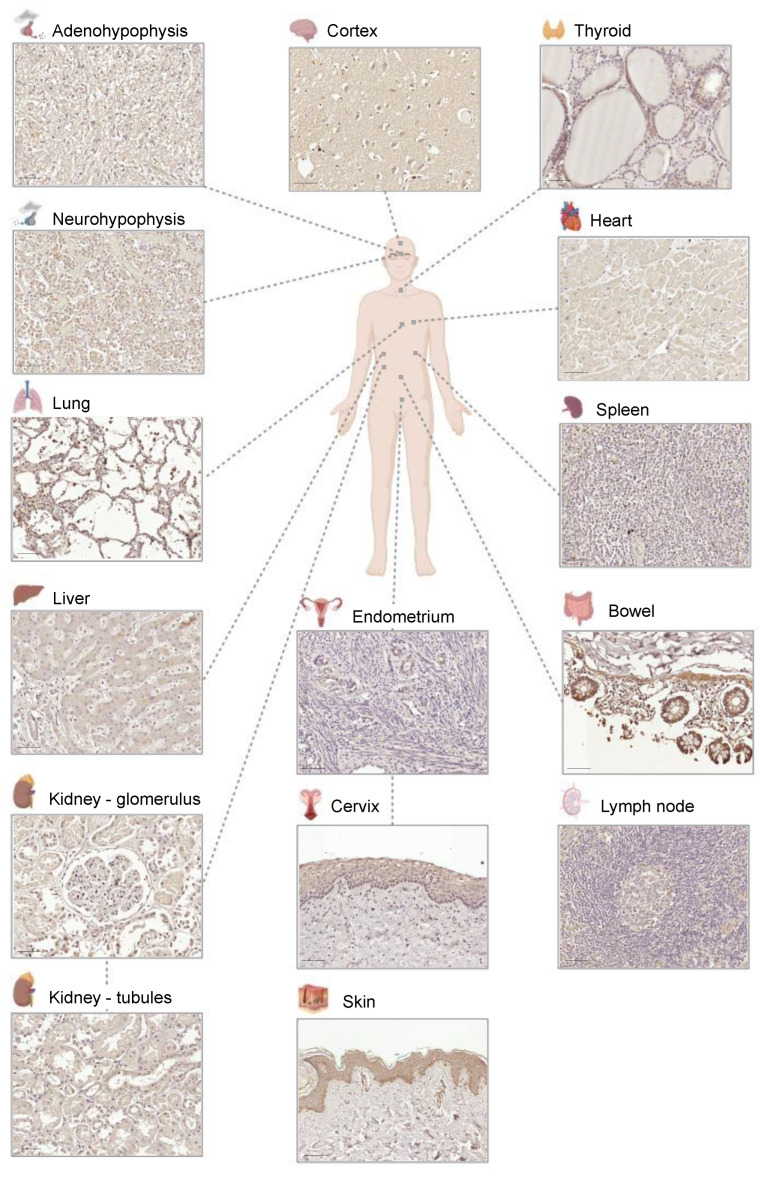
CSDE1 expression in healthy human tissues. IHC with the G10 monoclonal antibody is shown.

We next benchmarked the G10 antibody against 14 healthy tissues obtained from two patients and compared the staining patterns with those in the Human Protein Atlas (HPA) repository. Tissues included brain cortex, pituitary gland (adenohypophysis and neurohypophysis), thyroid, lung, heart, spleen, lymph node, liver, kidney, bowel, endometrium, cervix and skin (Figure 2). Except for adenohypophysis and neurohypophysis, the remaining tissues are present in the HPA repository. Staining patterns were in general concordant and provided clarification in cases where contradictions between existing antibodies were detected. For instance, the spleen is stained with only one of the HPA antibodies and our monoclonal detects weak uniform staining across the tissue.

We observed nearly ubiquitous presence of CSDE1 in tissues, although CSDE1 levels vary widely, with some tissues staining strongly (e.g., bowel) and others rather weakly (e.g., lymph node). Differences within tissues were also observed; for example, basal skin keratinocytes show strong perinuclear/nuclear staining of CSDE1, while keratinocytes in other strata display weaker and more diffuse staining, suggesting regulation of CSDE1 during keratinocyte differentiation. In several tissues, staining of CSDE1 is cytoplasmic, consistent with its molecular roles as regulator of mRNA translation and stability.

### 2.3. Global CSDE1 Levels Decrease with Melanoma Malignancy

**Figure 3:**
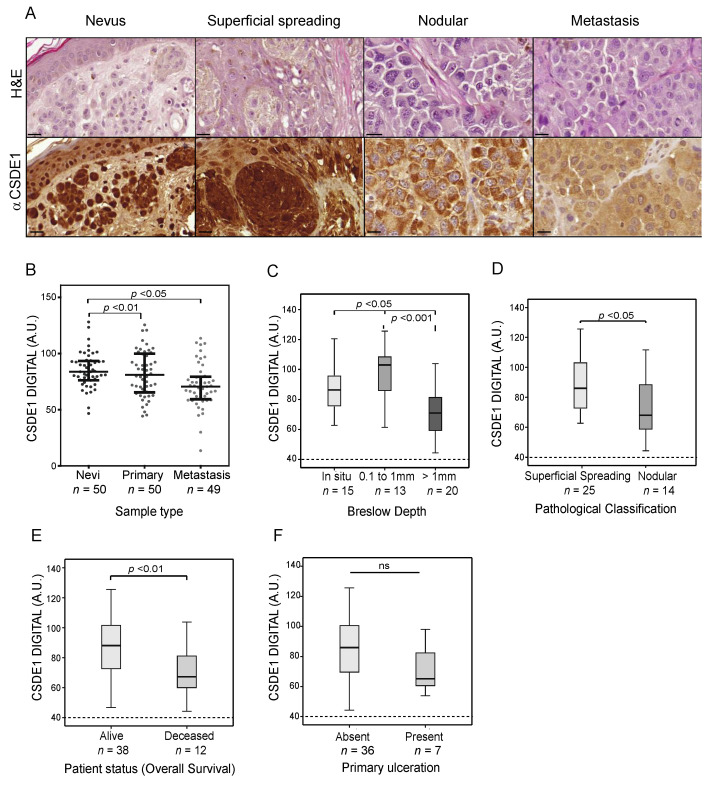
Reduced CSDE1 levels correlate with adverse clinical features. (**A**) Representative IHC images of nevi, primary and metastatic tumors. Hematoxylin and Eosin (H&E) staining is shown at the top and anti-CSDE1 staining at the bottom. Scale bar, 20 µm. (**B**) Global CSDE1 protein levels decrease with malignancy. Each dot represents the mean intensity of up to five fields within the same sample. (**C**–**F**) Correlation of CSDE1 levels in primary tumors with the indicated clinical features. Note that the Y-axis has been cut to emphasize differences (dashed line). Statistical significance was evaluated using one-way ANOVA with Bonferroni correction (**B**,**C**) or independent *t*-test (**D**–**F**).

The G10 antibody was then used to assess CSDE1 levels in nevi and melanoma samples. Images were quantified digitally as detailed in Materials and Methods, and CSDE1 levels were correlated with tumor parameters. Surprisingly, contrary with what would be expected for an oncoprotein, global CSDE1 levels decreased with malignancy, as they were high in nevi and lower in metastatic samples (Figure 3A,B). We further correlated CSDE1 levels in primary tumors with melanoma indicators of malignancy, such as Breslow depth and pathological classification (superficial spreading or nodular, NCCN melanoma guidelines V2.2020). We observed that CSDE1 expression was modestly but significantly lower in samples with Breslow >1mm and in nodular melanoma (Figure 3C,D). In addition, low levels of CSDE1 in primary tumors are associated with reduced overall survival (Figure 3E). No significant difference was found for other features of primary tumors, such as location (BANS, i.e., upper Back, Posterior Arm, posterior Neck and Scalp) and ulceration, although a trend towards lower CSDE1 levels was detected in ulcerated tumors (Figure 3F). Analysis of global CSDE1 levels within metastatic samples revealed no significant correlations (data not shown). In addition, despite a decrease in global levels with malignancy, no correlation was found between total CSDE1 staining and patient outcome in the examined cohort (data not shown). These results suggest that analysis of CSDE1 total protein levels may serve as an indicator of disease progression in the primary tumor setting.

### 2.4. CSDE1 Cytoplasmic/Nuclear Ratio Correlates with Disease-Specific Survival

**Figure 4:**
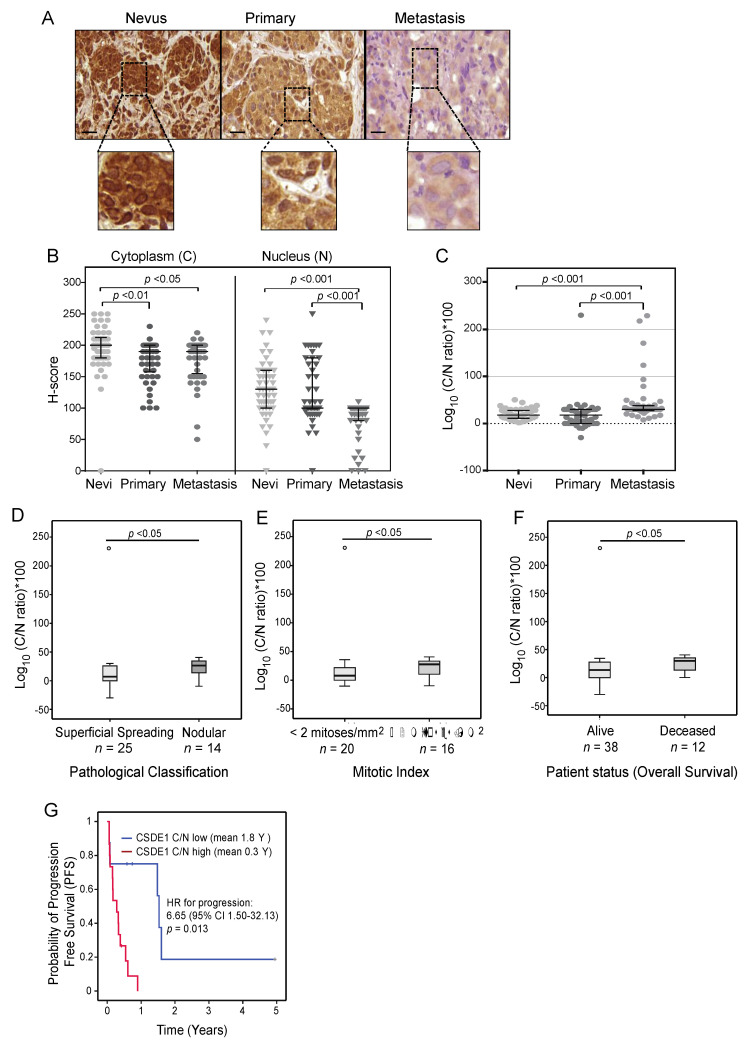
CSDE1 cytoplasmic/nuclear ratio is a marker of prognosis. (**A**) Representative images of nevi, primary and metastatic tumors, with augmented insets (dotted squares) to visualize intracellular distribution of CSDE1. Scale bar, 20 µm. (**B**) H-score quantification of CSDE1 levels in the nucleus and cytoplasm of cells in nevi, primary tumors and metastasis. (**C**–**F**) Cytoplasmic/nuclear CSDE1 ratios and correlation with the indicated clinical features of primary tumors. Log10 and *100 calculations have been applied to de-compact and better visualize the data on the graphical representations. (**G**) Kaplan–Meier curve of progression free survival of patients with primary cutaneous melanoma. Metastatic samples naïve for treatment with PFS data were included in the analysis (cut point = 1.93; *n* = 23; C/N low arm, *n* = 8; C/N high arm, *n* = 15). Statistical differences were evaluated using Kruskal–Wallis with Bonferroni correction (**B**,**C**), Mann–Whitney U (**D**–**F**) and Log Rank (**G**).

We noticed that CSDE1 can be found in both the cytoplasm and nucleus of melanoma patient samples depending on the sample type. CSDE1 nuclear staining was strong in benign nevi and superficial spreading melanoma, while the protein was primarily cytoplasmic in highly aggressive primary tumors (nodular) and metastatic lesions (see representative images in Figure 4A). We thus measured cytoplasmic (C) and nuclear (N) CSDE1 levels in our sample cohort and correlated the C/N ratio with clinical features. Because an automatic system to perform intracellular compartment quantification was not available to us, we used manual quantification employing the classical H-score and calculated the C/N ratio using the formula (1 + Cytoplasm H-score)/(1 + Nuclear H-score) (see Materials and Methods). Both the cytoplasmic and nuclear CSDE1 levels decreased with malignancy, although the decrease was more prominent for nuclear CSDE1 (Figure 4B). As a result, an increased C/N ratio was associated with metastasis (Figure 4C). No differences were found between primary melanoma and nevi, probably because a large fraction of our primary samples consist of superficial spreading melanoma.

However, significant differences were found among primary lesions. In primary melanoma, the CSDE1 C/N ratio increases in samples of nodular histology and with more than two mitoses (Figure 4D,E). Regarding outcome, the group of deceased patients had a significantly increased C/N ratio (Figure 4F). C/N ratios also positively correlated with BANS location of the primary tumor and ulceration, although in this case no significance was reached (data not shown). Importantly, patients with metastasis naïve for treatment derived from primary cutaneous melanoma showed a significant correlation between high C/N CSDE1 ratios and worse progression free survival (PFS), increasing the risk of PFS by 6.6 times (means of 1.8 years for low vs 0.3 years for high C/N) (Figure 4G). Taken together, these results indicate that an increased CSDE1 C/N ratio correlates with malignancy of primary melanoma lesions, and with poor outcome of patients with metastasis.

## 3. Discussion

In this study, we evaluate the prognosis potential of CSDE1 using a dedicated monoclonal antibody useful for IHC in the clinical setting. Contrary to expectations for an oncoprotein involved in invasion and metastasis, CSDE1 global levels decrease with malignancy. However, its intracellular distribution changes, correlating with adverse clinical features.

We show that the CSDE1 cytoplasmic/nuclear ratio is a marker of progression free survival. The partitioning of proteins between subcellular compartments has previously been proposed as a biomarker in cancer, and there is evidence about the potential of intracellular distribution of cell cycle inhibitors and transcription factors in prognosis (e.g., [28,29,30,31]). Regarding RBPs, the evidence is sparse, with perhaps the best characterized example being HuR. This protein binds to AU-rich elements (ARE) in the 3’ UTRs of targets involved in cell proliferation, differentiation and apoptosis and modulates their translation and stability [32]. HuR is primarily nuclear in resting cells, but it is modified and mobilized to the cytoplasm in response to mitogens and other stress signals [32]. Cytoplasmic HuR is elevated in many types of cancer and is associated with poor clinical outcome and therapy resistance, justifying current intense efforts to find HuR inhibitors for clinical applications [33,34]. CSDE1 thus adds to a yet small list of RBPs whose intracellular dynamics are modified upon oncogenic stimulation and correlate with prognosis. The biological rationale of CSDE1 nucleo–cytoplasmic distribution is currently unclear. Cytoplasmic functions of CSDE1 are important for tumor promotion, including the regulation of the synthesis of EMT factors resulting in increased invasiveness or the modulation of TCPTP mRNA stability leading to immune escape [19,20]. However, although most reported functions of CSDE1 are cytoplasmic, it has been recently proposed that CSDE1 interacts with RNApol-II and CDK7 to promote transcription in breast cancer cells [35]. Furthermore, the *Drosophila* homologue of CSDE1 enhances the assembly of the dosage compensation complex on the X-chromosome leading to hyper-transcription [36]. CSDE1 nuclear levels clearly decrease with malignancy. Thus, CSDE1 may actively function on nuclear processes to preserve cell homeostasis or may be simply confined in the nucleus as a protective mechanism against cell transformation. Intriguingly, CSDE1 is mostly cytoplasmic in healthy tissues, suggesting that cytoplasmic localization per se is not the only factor underlying the oncogenic functions of CSDE1.

In addition to CSDE1, several RBPs have been shown to play roles in melanoma progression, including CELF, CPEB4, IGF2BP1, IGF2BP3, NOVA1 and DDX3X, among others [37,38,39,40,41,42]. It will be interesting to decipher their potential as melanoma biomarkers and their synergies with CSDE1.

CSDE1 has been proposed as a predictive marker for anti-PD1 immunotherapy response in melanoma and as a prognostic biomarker helping to guide adjuvant treatment decisions in resectable pancreatic adenocarcinoma [20,22]. These findings, along with the outcomes of our study, highlight the robust potential of CSDE1 as a predictive marker for patient outcome. Future investigations, analyzing CSDE1 levels and intracellular distribution in focused patient cohorts, will unveil the full value of CSDE1 as a biomarker and potential therapeutic target. Of note, a recent study revealed the antibiotic clofoctol as a drug targeting CSDE1 for the treatment of glioblastoma, placing CSDE1 in the cancer druggable space [43]. Altogether, the results suggest a promising future for CSDE1 in the medical arena.

## 4. Materials and Methods

### 4.1. Ethical Statement

Samples were obtained from Hospital Germans Trias i Pujol (HGTP, Badalona, Spain) after approval of this study by the Clinical Ethics Committee of HGTP. Project ID code: BB19018, date of approval: 14 June 2019. This study was conducted in accordance with the Declaration of Helsinki, and all subjects gave their informed consent for inclusion before they participated in this study.

### 4.2. Immunohistochemistry (IHC) of Human Tissue Specimens

IHC was performed on 5 mm thick sections of formalin-fixed paraffin-embedded sample (FFPE) blocks (complete histological sample) at the ICTS Nanbiosis FVPR Unit of VHIR. Samples were dewaxed in a stove at 60 °C for 30–60 min, incubated 3 times in Xylol for 5 min and hydrated using serial immersions of 5 min in ethanol (100%, 96%, 70%, and 50%), with a final wash in distilled water for 5 min. Antigen retrieval was performed in 0.1M citrate buffer (DAKO) in a microwave (750 W for 15 min followed by 450 W for 15 min, keeping the sample wet in buffer). After a first step of blocking endogenous peroxidase with 3% H_2_O_2_ for 10 min, and a second step of blocking nonspecific antibody binding with 10% normal goat serum (Vector Lab) in TBS (150 mM NaCl and 50 mM Tris-HCl pH 7.6) + 1% BSA for 10 min; immunohistochemical staining of CSDE1 was performed using a custom-made anti-CSDE1 mouse monoclonal antibody (see Results) at a final concentration of 20 ng/µL overnight at 4 °C. Samples were rinsed with TBS, incubated with EnVisionTM HRP Rabbit/Mouse Labelled Polymer (DAKO) for 30 min at room temperature (RT) and revealed with 3,3′-diaminobenzidine (DAB) for 5 min at RT. Slides were counterstained with hematoxylin, dehydrated in serial ethanol solutions and xylol and mounted in DPX.

### 4.3. Immunocytochemistry (ICC)

SK-Mel-147 cells were washed with PBS and fixed with 10% formalin solution (Sigma HT501128) for 3 h at 4 °C. Cells were then washed twice with PBS and embedded in paraffin. ICC was performed at the ICTS Nanbiosis FVPR Unit of VHIR using the same protocol as for IHC. Antibodies were used at the following final concentrations: G10 (20 ng/µL) and ab96124 (15 ng/µL).

### 4.4. Visual and Digital Quantification

Glass slides were scanned using a 3DHISTECH scanner (Pannoramic 250) at 20× equivalent magnification using automatic and manual detection. The CaseViewer software was used to evaluate and select up to five representative areas from each sample and calculate the mean intensity. Areas with strong melanin staining were avoided and were thus not selected in a blinded manner. Digital quantification was performed using Andy’s Algorithms employing FIJI [44]. Visual quantification was performed using the H-Score method. The H-score was defined using a scale of 0 (no staining), 1 (weak), 2 (moderate) and 3 (strong) for all measurable tissues. After determining the intensity in all samples, the following formula was applied: H-score = [1 × (% cells 1)] + [2 × (% cells 2)] + [3 × (% cells 3)] [45]. The cytoplasm/nuclear ratio (C/N ratio) was calculated using the formula (1 + Cytoplasm H-score)/(1 + Nuclear H-score). Raw measures were processed adjusting their scale (255-Raw measure) and subtracting the signal from negative controls consisting of stainings without primary antibody.

### 4.5. Statistical Analysis

All statistical analyses were performed using IBM SPSS statistics 22, R studio and GraphPad software. Statistical methods used were one-way ANOVA, Mann–Whitney U, Kruskal–Wallis and Student’s t-test depending on the particular comparison, as indicated in the figure legends. Normality tests were conducted in order to determine the most suitable statistical method for each comparison.

In dot plot graphs, each dot represents the value from one sample, the middle bar is the median and the upper and lower bars represent the interquartile range (25th to 75th). In box plot graphs, the middle line represents the median, the bottom and the top of the box indicate the 25th and 75th percentile, respectively, and the whiskers extend to 1.5 times the height of the box or, if no case/row has a value in that range, to the minimum or maximum values. The points indicate the outliers.

Survival curves were made using the Kaplan–Meier method. The best cut point was obtained using Cutoff Finder, X-tile and ROC curve in SPSS programs. First, Cutoff Finder and X-tile were used to identify the most promising clinical feature correlating with CSDE1 data (PFS). Then, the ROC curve method was applied to PFS data to test for specificity and sensitivity of the CSDE1 C/N ratio as a biomarker. A cutoff of 1.93 was chosen, yielding a sensitivity of 73.7% and specificity of 75%. The log-rank test was used to compare survival groups and Cox regression was performed to assess the contribution to clinical or molecular features.

### 4.6. Cell Culture and Depletion

Melanoma SK-Mel-147 cells were grown at 37 °C in Dulbecco’s Modified Eagle Media (DMEM) supplemented with pyruvate (Invitrogen), 10% fetal bovine serum (FBS) and 1% penicillin–streptomycin. CSDE1 was depleted with siRNA pools (siTools Biotech) following the recommendations of the vendor or with shRNA via treatment of shCSDE1 or shControl expressing cells with 0.5 µg/mL doxycycline, as previously described [19].

## Figures and Tables

**Table 1:** Clinical features of primary tumor samples.

Feature		*n* (%)
Gender	Male	25 (50)
	Female	25 (50)
Age (years)	<58	11 (22)
	≥58	39 (78)
Location	Face, scalp or neck	9 (18)
	Trunk	19 (38)
	Upper extremities	6 (12)
	Lower extremities	13 (26)
	NA *	3 (6)
Histopathological subtype	Superficial spreading	25 (50)
	Nodular	14 (28)
	Other (in situ, acral, lentigo, mixed histology and nonclassifiable)	11 (22)
Breslow	In situ	15 (30)
	<1 mm	13 (26)
	≥1 mm	20 (40)
	NA	2 (4)
Ulceration	No	36 (72)
	Yes	7 (14)
	NA *	7 (14)
Mitotic index	<2 mitoses/mm^2^	20 (40)
	≥2 mitoses/mm^2^	16 (32)
	NA *	14 (28)

* NA, not available.

**Table 2:** Clinical features of metastatic samples.

Feature		*n* (%)
Gender	Male	32 (65)
	Female	17 (35)
Age (years)	<58	13 (27)
	≥58	36 (73)
Primary type	Cutaneous	40 (82)
	Noncutaneous	3 (6)
	Unknown primary	6 (12)
Metastasis type	Cutaneous	16 (33)
	Lymph node	21 (43)
	Visceral	12 (24)
Molecular subtype	BRAF	20 (41)
	NRAS	7 (14)
	WT	9 (18)
	NA *	13 (27)
Stage at diagnosis	Stage I	4 (8.2)
	Stage II	12 (24.5)
	Stage III	22 (44.9)
	Stage IV	6 (12.2)
	NA *	5 (10.2)
LDH at diagnosis	Normal	11 (22.5)
	Increased	5 (10.2)
	NA *	33 (67.4)
Treatment before sample collection	Naïve	38 (78)
	Interferon	8 (16)
	Other	3 (6)

* NA, not available.

## Data Availability

The raw data supporting the conclusions of this article will be made available by the authors on request.

## Supplementary Materials

The following supporting information can be downloaded at: https://www.mdpi.com/article/10.3390/ijms25042319/s1.
