# Supplementary material for: CSDE1 Intracellular Distribution as a Biomarker of Melanoma Prognosis
Source: Int J Mol Sci. 2024 Feb 15;25(4):2319. doi: 10.3390/ijms25042319 (PMC10889260; doi:10.3390/ijms25042319)
Supplement: Supplementary file 1 [file ijms-25-02319-s001.zip › ijms-2761391-supplementary.pdf]

Figure S1- Indacochea et al

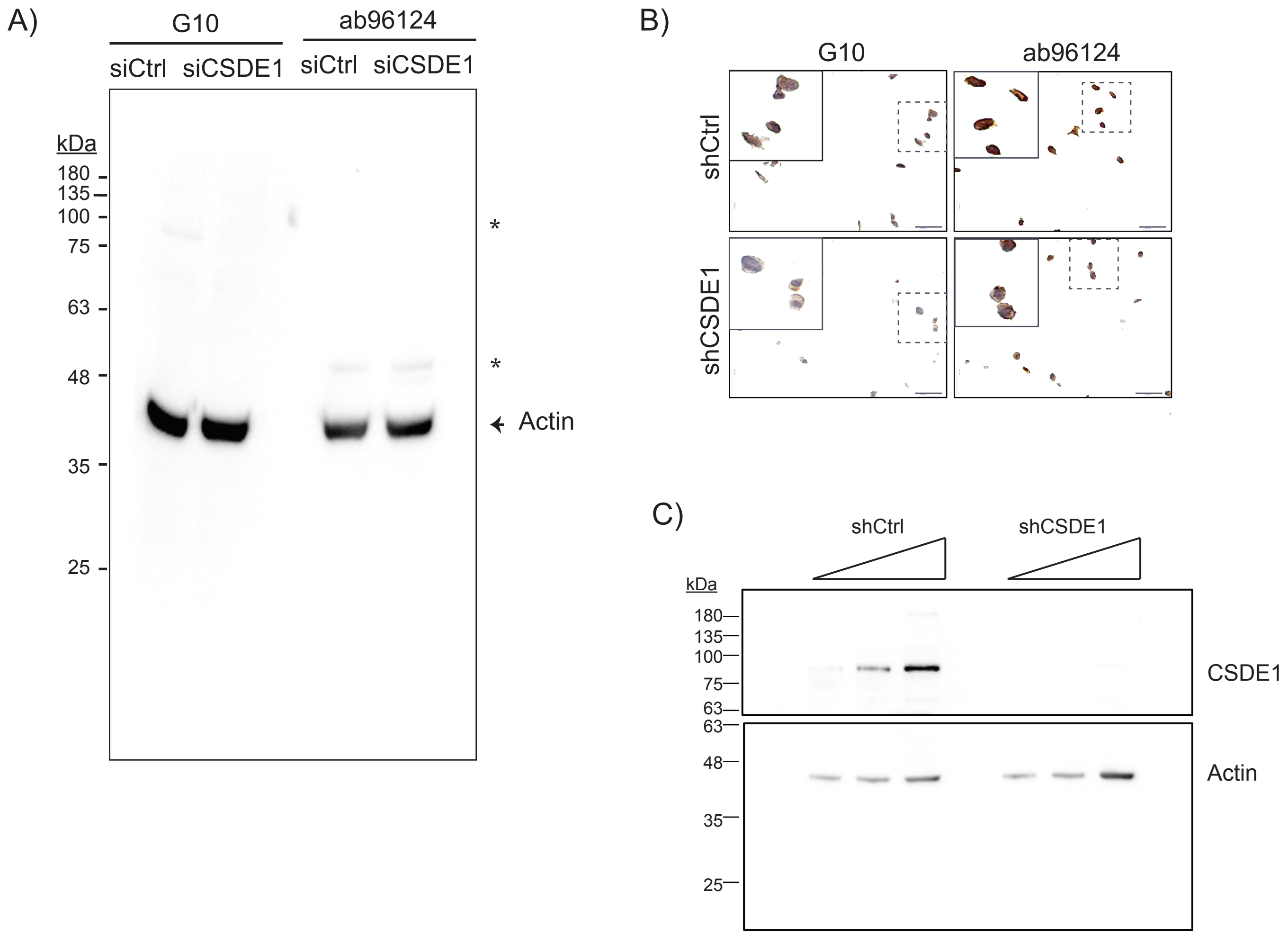


**Figure S1**. Specificity of CSDE1 antibodies. A) Full blots of actin (related to Figure 1A). Asterisks denote residual signal from prior Western blot with anti-CSDE1 antibodies. B) Immunocytochemistry of melanoma SK-Mel-147 cells with the G10 and ab96124 antibodies. Cells treated with shRNA against CSDE1 (shCSDE1) or shRNA control (shCtrl) are shown. Higher magnification images are shown in the insets. Scale bar, 50 $\mu$m. C) Full blots showing the efficiency of CSDE1 depletion of cells used in (B). Increasing amounts of cell extracts were used for Western
